# A Double-Blind, Placebo-Controlled Study of the Aqueous Extract of *Echium amoenum* for Patients with General Anxiety Disorder

**Published:** 2012

**Authors:** Mehdi Sayyah, Amir Siahpoosh, Hamidreza Khalili, Alireza Malayeri, Hamidreza Samaee

**Affiliations:** a *Herbal Medicine Research Center, Jundishapur University of Medical Sciences, Ahwaz, Iran.*; b *Department of Pharmacology, Jundishapur University of Medical Sciences, Ahwaz, Iran.*; c *College ofPharmacy, Jundishapur University of Medical Sciences, Ahwaz, Iran.*

**Keywords:** Clinical trial, Echium amoenum, General anxiety disorder, Aqueous extract

## Abstract

The aim of this study was to assess the efficacy and tolerability of the aqueous extract of *Echium amoenum* in combination with SSRIs in patients with General Anxiety Disorder (GAD). The study was an 8-week double-blind randomized clinical trial. Thirty-seven adult outpatients who met the DSM-IV-TR criteria for GAD based on the structured clinical interview participated in the trial. In this study, patients were randomly assigned to receive the aqueous extract (500 mg) plus fluoxetine or fluoxetine (20 mg/day) plus placebo. The results showed significant difference between the two groups in the treatment of GAD. Moreover, there was not any significant difference between the two groups in terms of observed side effects. *E. amoenum* is effective on anxiety disorder, especially in higher dosage, without any serious side effects.

## Introduction

General Anxiety Disorder (GAD) is characterized by excessive worry and anxiety that are difficult to control and cause significant distress and impairment. *Echium amoenum *or Boraginaceae is a wild annual herb which is known in Iran as Ox-tongue (1, 2). The plant grows in the northern mountains of Iran (2) and has been recommended for mood enhancement (3). The plant is traditionally either brewed or boiled in water before drinking (4). The benefits of this traditional medication have initially been discovered by the Romans, 300 B.C. (4).

The extract contains flavonoids, saponins, unsaturated terpenoids and sterols (5). St. John’s Wort, also contains flavonoids and is thought to exert its effect through them (6). Therefore, the presence of flavonoids in the *E. amoenum* extract may be due to the anti anxiety effect of the plant. In a recent study, aqueous extract of *E. amoenum* flower was administered on animals and the results showed that the extract had anxiolytic effects (7). The study indicated that the extract was effective at intraperitoneal doses of 80-125 mg/Kg and toxicity did not occur with doses as high as 6 g/Kg (7). A human study has also shown that the aqueous extract of *E. amoenum* flower had antidepressant effect (8) .The results of another human study showed that 500 mg/day aqueous extract of *E. amoenum* had positive effects on the obsession and compulsion, generalized without any side effects (9).

Therefore, the authors decided to examine the efficacy and safety of Ondansetron in the treatment of GAD.

## Experimental

*Participants and setting*

Participants were eligible for the study if they met DSM-IV-TR criteria for GAD (10) and if they had scored 18 or above in the Hamilton anxiety rating scale of 14 items (HAM-A_14_) (11). After the diagnoses, in order to investigate the effectiveness of treatment, the HAM-A_14_ was administered once more. To enter the study, patients had to score 18 or higher in the HAM-A, be between 18 and 60 years old and give written informed consent for participation in the study. In addition, they should not have received any other psychiatric medication during the past six weeks prior to the beginning of the study. Subjects were disqualified if they had any psychotic symptoms or suicidal thoughts and if they had any other psychiatric or neurological disorder. The patients were also assessed for significant cardiac, renal or hepatic diseases, pregnancy, lactation, and mental retardation. The presence of any of these conditions excluded the patients from participating in this study. The study was conducted from August, 2009 to June, 2010 march, in the outpatient clinic of Imam Khomeini General Hospital, Ahwaz, Iran. Patients could be withdrawn from the study at any time during the trial and transferred to a conventional treatment. Those completing the trial were also guaranteed to be transferred to a conventional treatment as well. The study was conducted in accordance with the Declaration of Helsinki and Tokyo for humans and was approved by the Ethics and Research Committee at Jundishapur University of Medical Sciences.

*Preparation of medication*

The fresh flowers of *E. amoenum *were collected from the northern mountains of Iran in May 2005. *E. amoenum *was authenticated by M. K. and deposited in voucher No. 628 in the herbarium of the Faculty of Pharmacy at Shahid Beheshti University of Medical Sciences. In traditional medicine, *E. amoenum *is brewed before the use, so, we used aqueous in this study. Each 10 mg of air-dried flowers of the plant was boiled with 300 mL water for 15 min. The mixture was then filtered and the filtrate was concentrated with water bath at 37°C. Each 10 mg of the dried flowers yielded to 4.6 g of aqueous extract. In order to preserve the double blind condition, *E. amoenum *extract and placebo were dispensed in identical-appearing capsules. *E. amoenum *capsules were filled with 250 mg of the extract and talcum powder while placebo capsules were only filled with talcum powder.

*Procedures*

After obtaining the written informed consents from the patients or guardians, participants entered either of the parallel groups using a computer-generated list of random numbers. Patients were randomly assigned to be treated either with the Extract of E*. amoenum *(750 mg daily), all patients received one oral capsule 3 times a day [one capsule in the morning (8-9 a.m.) and afternoon (13-14 p.m.) and one capsule at nights (22-23 p.m.)] plus fluoxetine (20 mg/day) or Placebo plus fluoxetine (20 mg daily). Fluoxetine capsules were used from Dr. Abidi’s pharmaceutical and their brand names were Oxetine ®. Placebo was prepared by herbal medicine research center of Jundishapur university medical sciences with using from number 2 hard gelatin capsule for prepared Placebo. No other psychotropic medication was administered. Participants did not receive any concomitant psychological therapy or support. The participants in this study had not been using any psychiatric medication or therapy from six weeks prior to the beginning of the study. The patients were examined by psychiatrist before the trial and during the days 14, 28, 42 and 56. Effectiveness of the treatment was assessed using HAM-A_14_. The evaluation of patients was done with the psychiatrist. Treatment-induced adverse effects were assessed systematically at each visit by a score sheet designed specifically for the study. The patients were assured that in the case of any severe and uncontrollable side effects including agitation and headache, they could be withdrawn from the study and be treated for the side effects and their GAD will also be treated using standard procedures. The researchers had also agreed upon giving oxazepam 10 mg during the study in the case of experiencing insomnia by the patients.

**Figure 1:**
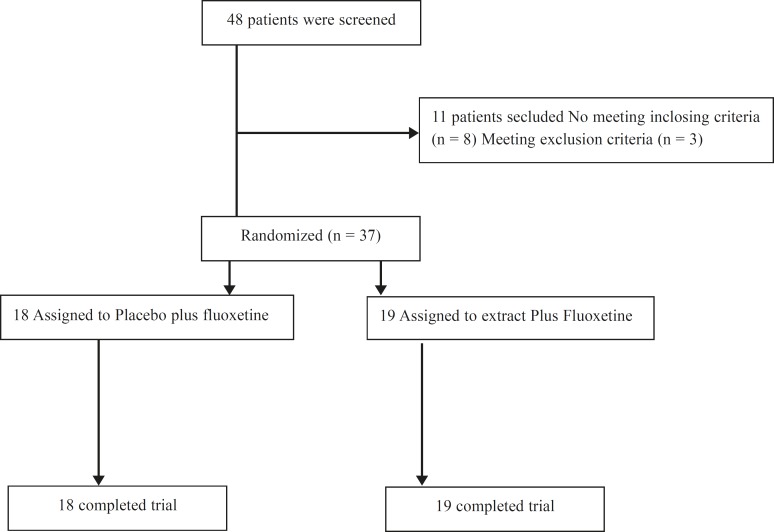
CONSORT diagram showing the disposition of all subjects screened for the study.

*Statistical analysis*

In this assay, a two-way repeated measures analysis of variance (time-treatment interaction) was used. The two groups as a between subjects factor (group) and the eight weekly measurements during treatment as the within-subjects factor (time) were considered in this trial. This was done for HAM-A_14_ total scores. In addition, a one-way repeated measures analysis of variance with a two-tailed post-hoc Tukey mean comparison test were performed in the change from baseline for HAM-A_14_ scores in each group. To compare the two groups at baseline and the outcome of two groups at the end of the trial, an unpaired Student›s t-test was used with a two-sided p-value. Results are presented as mean ± SEM and differences were considered significant with p ≥ 0.05. To compare the demographic data and the frequency of side effects between the protocols, Fisher›s exact test (two-sided) was performed. The authors used SPSS (version 24) for statistical analysis.

*Side effects*

Side effects were systematically recorded throughout the study and were assessed using a checklist administered by the psychiatrist of the study on days 0, 14, 28, 42, and 56.

## Results and Discussion

*Demographic characteristics and attrition*

Thirty-seven patients enrolled in the study; 19 were assigned to the *E. amoenum *group and 18 to the placebo group (Figure 1). Both groups had no statistically significant differences (Table 1).

*Efficacy: fluoxetine plus E. amoenum versus fluoxetine plus placebo*

**Figure 2:**
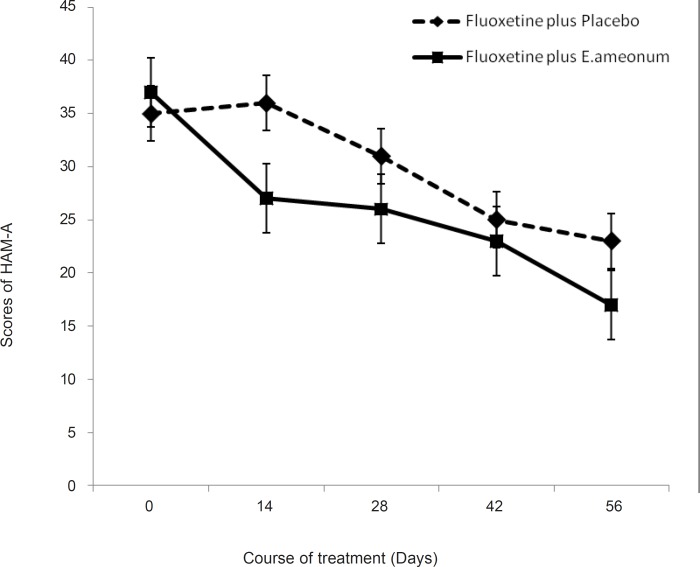
Effect of E. amoenum plus fluoxetine on score of Hamilton anxiety scale-14 items (HAM-A_14)_. Each point represents mean ± SD for 19-18 patients.*: p < 0.05.

As shown in Figure 2, the mean HAM-A scores gradually declined in the both study groups during the trial. ANOVA revealed a significant effect of time (F = 37.4, p < 0.01). The effect of treatment was significant (F = 2.12, p = 0.045). Time-by-treatment interaction was significant (F = 4.7, p = 0.013). The difference between the two treatments was significant after 14 days (t = 1.32, d.f. = 41, p = 0.00) and end point (t = 1.01, d.f. = 38, p = 0.018).

**Table 1:** Demographic data of the participants.

**Characteristic**	**Fluoxetine + Placebo** **Group (18)**	**Fluoxetine + ** ***E. ameonum*** **Group (19)**	**p**
Sex	Female : 9	Female : 9	0.19
	Male : 9	Male : 10	0.21
Marital status	Married : 8	Married : 9	0.41
	Single : 10	Single : 10	0.18
Age (Years)	Mean (SD) 26 ± 2.65	Mean (SD) 25 ± 3.76	0.24

*Clinical complications and side effects*

The difference in the frequency of side effects between the fluoxetine plus *E. amoenum* and fluoxetine plus placebo was not significant. Patients receiving the extract did not show more adverse effects than those taking the placebo.

The most common adverse effect in the extract group was headache (p = 0.41).

The results of this study show that the *E. amoenum *may have positive effects on the anxiety and it seems that the positive effects start from the second week. In addition, the results did not show any serious side effects accompanying the *E. amoenum*. These findings are in accordance with the ancient medical literature (especially Makhzan-Al-Adviye written by Khorasani and Canon of Medicine written by Avicenna). The limitation of this study was small sample size. Further studies with larger sample size are needed to verify the results of this study.

In previous studies that evaluated the effects of this plant on humans, a dosage of 375 mg/day and 500 mg were used to prevent possible side effects and no side effects were reported by this dosage (8, 9). The dosage in this study was 250 mg higher than previous ones. By this dosage (750 mg/day) no severe side effects were seen in the experimental group. In previous study (8) with dosage of 375 mg/day, there was not any treatment effect on anxiety but in dose of 500 mg/day (9), the medicine was effective on anxiety and obsessive-compulsive disorder.
